# Incidence of lumbar spondylolysis in athletes with low back pain: A systematic evaluation and single-arm meta-analysis

**DOI:** 10.1097/MD.0000000000034857

**Published:** 2023-09-22

**Authors:** Jingyuan Li, Jinlong Liang, Yongqing Xu, Di Du, Fanzhe Feng, Junhong Shen, Yi Cui

**Affiliations:** a Clinical Medical College of Dali University, Dali, China; b Department of Orthopaedic Surgery, 920th Hospital of Joint Logistics Support Force, Kunming, China.

**Keywords:** adolescent, lumbar isthmic fracture, prevalence, retrospective analysis

## Abstract

**Background:**

Low back pain (LBP) is a common chief complaint from athletes. Lumbar spondylolysis (LS) is a common sport injury. Severe LS is likely to cause spinal instability, resulting in lumbar spondylolisthesis or lumbar disc herniation, and even damage to the spinal nerve roots. The incidence of LS is approximately 5% in the adult population, and nearly half of young athletes with LBP are diagnosed with LS. This meta-analysis analyzed the incidence of LS in athletes with LBP.

**Methods:**

PubMed, Embase, Cochrane (Cochrane Central Register of Controlled Trials), and Web of Science databases were systematically searched for published case report and retrospective analyses related to the topic from the date of database creation to January 1,2023. Relevant literature was screened and information extracted, and risk of bias was assessed for included studies using the methodological index for non-randomized-studies scale. Single-arm Meta-analysis was performed using R4.04 software. Heterogeneity was quantified by Cochran *Q* test and Higgins *I*^2^. Funnel plots were used to visualize publication bias, and Egger test and Begg test were used to statistical tests.

**Results:**

A total of 9 studies (835 patients) were included in this study. Meta-analysis revealed that the prevalence of LS in athletes with LBP was estimated at 41.7%, [95% CI = (0.28–0.55)], but this prevalence varied considerably with the gender and age of the athletes.

**Conclusion:**

The estimated prevalence of LS in athletes with LBP is 41.7%, and future correlations between the prevalence of LS in adolescent athletes worldwide need to be assessed from different perspectives, including biomechanical, hormonal, anatomical, behavioral, and gender differences.

## 1. Introduction

Low back pain (LBP) is a common chief complaint from athletes. It is reported that the incidence of LBP in athletes is much higher than that in nonathletes.^[1]^ More than 30% of athletes developed LBP during training. About 75% of elite athletes had a history of 1 or more onset of LBP.^[2–4]^ LBP may be caused by many factors such as anatomy, sports, psychology, and society. Micheli and Wood have reported that lumbar spondylolysis (LS) is the most common cause of diagnosable LBP.^[4–6]^ Brinjikji et al^[7]^ have reported that spondylolysis is more prevalent in young adults (< 50 years old) with back pain compared with asymptomatic individuals. LS is defined as discontinuity in the pars interarticularis defect between the upper and lower articular processes on 1 side or both sides of vertebral arches of the lumbar spine. This injury is pars fracture or defect in the spine. This injury occurs most often at L5, followed by L4.^[8]^ Severe LS is likely to cause spinal instability, resulting in lumbar spondylolisthesis or lumbar disc herniation, and even damage to the spinal nerve roots. The incidence of LS is approximately 5% in the adult population, and nearly half of young athletes with LBP are diagnosed with LS.^[9]^ This meta-analysis analyzed the incidence of LS in athletes with LBP so as to understand the correlation between the LS and LBP and promote early prevention and timely treatment. We have registered this meta-analysis on the INPLASY website (https://inplasy.com) [DOI number: 10.37766/inplasy2023.4.0006].

## 2. Materials and methods

### 2.1. Search strategy

We systematically searched PubMed, Embase, Cochrane, and Web of Science databases for relevant studies published up to May 1,2022. The search was conducted in the form of medical subject headings (MeSH) + free words, and the PubMed search terms were Low Back Pain [Mesh], Athletes [Mesh], and lumbar spondylolysis [Mesh], and the detailed search strategy is shown in the Supplemental Digital Content 1, http://links.lww.com/MD/J538. In addition, we conducted a manual search of the bibliographies of previous systematic reviews and relevant randomized controlled trials to retrieve additional studies that may not have been identified in the electronic search. This meta-analysis strictly follows the preferred reporting item guidelines for systematic reviews and meta-analyses.^[10]^

### 2.2. Inclusion and exclusion criteria

The aim of this study was to analyze the incidence of LS in athletes with concomitant LBP, for which we developed the following inclusion and exclusion criteria.

#### 2.2.1. Inclusion criteria.

1. The study population was athletes who had a history of lower back pain and;
2. The outcome indicators included the incidence of lumbar isthmic fractures.

#### 2.2.2. Exclusion criteria.

1. Reviews, case series involving fewer than 10 patients, case controls, etc.
2. Study population discrepancy or small sample size (sample size is <30).
3. Disease discrepancy.
4. Outcome indicators discrepancy.

### 2.3. Literature screening and information extraction

We imported the retrieved studies into EndnoteX9, filtered by title and abstract after automatic duplicate search and manual elimination of duplicates and studies, and downloaded the remaining eligible studies and filtered them according to full text. We extracted the following data from the included studies using standardized tables: title, first author, year of publication, author country, study type, gender, age, and type of exercise. Outcome indicators include the number of occurrences of lumbar septal fractures and the total number of cases. The above literature screening and information extraction were performed independently by 2 reviewers, cross-checked after completion, and a third investigator was asked to assist in ruling if there was a dispute.

### 2.4. Quality evaluation

Two independent risk of bias assessments were performed using the methodological index for non-randomized-studies scale (methodological index for non-randomized-studies)^[11]^ for the inclusion of non-randomized controlled interventional studies. The assessment consisted of 12 evaluation indicators, each scored 0 to 2, the first 8 for studies without a control group, with a maximum score of 16; A score of 0 indicates that it was not reported; a score of 1 indicates that it was reported but with insufficient information; and a score of 2 indicates that it was reported and sufficient information was provided. Two researchers cross-checked after the assessment was completed, and if there was disagreement, a third researcher assisted in the ruling.

### 2.5. Data analysis

R4.04 software (R Development Core Team, Vienna, http://www.R-project.org) was used to perform the single-arm Meta-analysis, in which the metafor package, the matrix package and the meta package were used. Heterogeneity was quantified statistically by Cochran Q test and Higgins *I*^2^. The different cutoff intervals of *I*^2^ values from 0 to 25%, 26% to 50%, 51% to 75%, and 76% to 100% correspond to no significant, moderate, significant and very strong heterogeneity, respectively. When *I*^2^ < 50% then the fixed effects model was used to combine the effect sizes. When *I*^2^ > 50%, effect sizes were combined using a random-effects model, and sources of heterogeneity were explored using subgroup and sensitivity analyses. Funnel plots were used to visualize publication bias, and statistical tests were performed using Egger test and begg test. The difference was statistically significant at *P* < .05.

## 3. Result

### 3.1. Literature retrieval results

**Figure 1.**
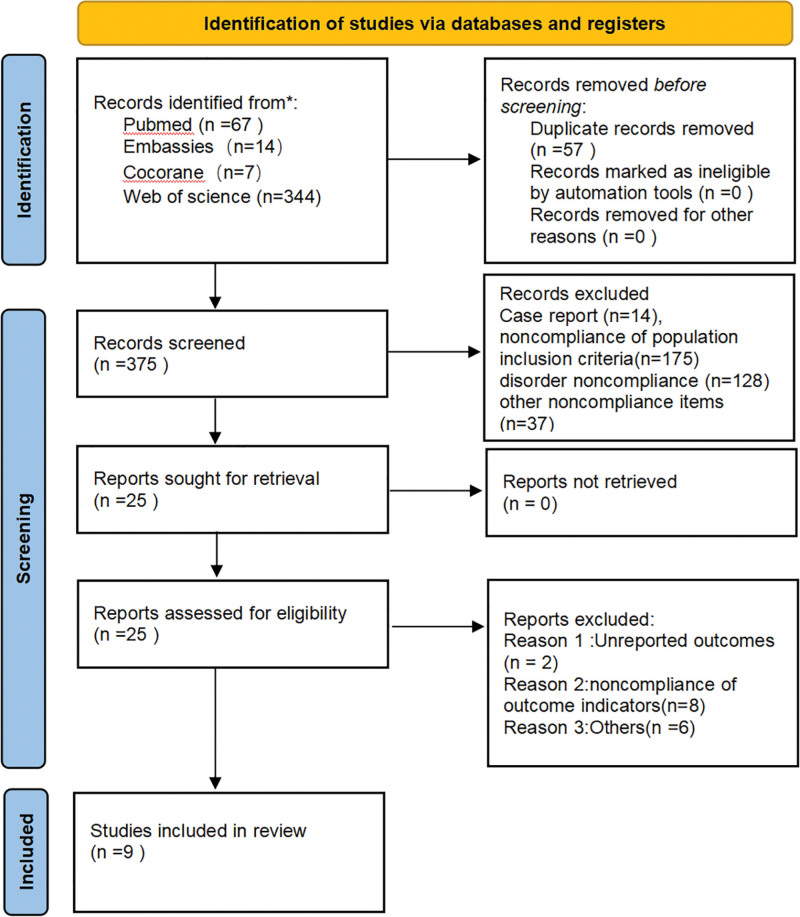
Flow chart of literature search.

Our electronic search strategy and study selection flowchart is shown in Figure 1, searching a total of 432 relevant publications (Pubmed (n = 67) Embase (n = 14) Cochrane (n = 7) Web of science (n = 344)). Case report category (n = 14) was screened after removing duplicates (n = 57), population inclusion criteria did not match (n = 175) disease did not match (n = 128) other nonconformities (n = 37). We identified 25 studies that met the initial screening requirements, of which we performed a full-text close reading, of which 8 did not meet the outcome indicators and 2 did not complete the trial, leaving 9 studies (835 patients in total) eligible for inclusion in our systematic evaluation and meta-analysis after removing other ineligible items (n = 6). The screening process is shown in Figure 1.

### 3.2. Basic characteristics of the included literature

The studies included in this article were published between 2005 and 2021, with patients mainly from Japan, the United States and the United Kingdom. A total of 503 men and 236 women (individual studies did not distinguish between the sexes of the athletes). Sports include aerobic sports such as long-distance running, swimming, rope skipping, and competitive sports such as basketball, soccer, volleyball, pole vaulting, and weightlifting. The average age of the athletes was 16.1. The occurrence of lumbar isthmic fractures is mainly distributed in the L4 and L5 segments. The specific information is shown in Table 1.

**Table 1:** Table of basic characteristics of the included literature.

	Author	Year	Country	Study design	Gender (male/female)		Age (mean)	Sport	Inc	Total	The site of pain
1	Peter L	2005	UK	Large retrospective case series.	131	82	17.3	Aerobic exercise	81	213	L1-3: 16% L4–5: 84%
2	J.Iwamoto	2005	Japan	A prospective study	-	-	15.5	Competitive sports	26	96	-
3	Kobayashi A	2013	Japan	A prospective cohort design	144	56	14.1	Aerobic exercise	97	200	L2-3: 35% L4–5: 65%
4	Donaldson LD.	2014	USA	Retrospective case review.	25	0	16.5	Competitive sports	11	25	L2-3: 45% L4–5: 65%
5	Schroeder GD.	2016	USA	A retrospective review	27	39	15.1	Aerobic exercise	32	66	-
6	Yamashita K	2019	Japan	A prospective study	54	15	15.2	Aerobic exercise	51	69	-
7	Yamashita K (2)	2019	Japan	Case series	16	7	30.4	Aerobic exercise	1	23	-
8	Takuji Y	2021	Japan	Cohort study	85	37	15.1	Aerobic exercise	75	122	-
9	Enoki S	2022	Japan	A retrospective review	21	0	22.2	Competitive sports	6	21	L2-3: 0 L4–5: 100%

### 3.3. Quality evaluation of included literature

We used the methodological index for non-randomized-studies scale (methodological index for non-randomized-studies)^[11]^ to assess the risk of bias for the inclusion of non-randomized controlled interventional studies. There was no control group in this study so the risk of bias for the first 8 items of the assessment scale was assessed with a maximum score of 16; a score of 0 indicated not reported; a score of 1 indicated reported but with insufficient information; and a score of 2 indicated reported and sufficient information was provided. The evaluation results are shown in Table 2.

**Table 2:** Quality evaluation table of included literature.

Author	Year	v1	v2	v3	v4	v5	v6	v7	v8
Peter L	2005	1	2	2	1	2	2	2	0
J.Iwamoto	2005	2	2	1	2	2	1	2	0
Kobayashi, A.	2013	2	2	2	2	2	1	2	0
Donaldson LD.	2014	2	1	1	2	2	2	2	0
Schroeder GD.	2016	2	2	1	1	2	2	2	0
Yamashita K	2019	1	2	1	1	2	1	2	0
Yamashita K (2)	2019	1	2	1	1	2	1	2	0
Takuji Y	2021	2	1	1	1	2	2	2	0
Enoki, S	2022	2	2	1	1	2	1	2	0

Note: V1: the purpose of the study was clearly given. V2: the continuity of included patients. V3: Expected data collection. V4: endpoint indicators appropriately respond to the purpose of the study. V5: objectivity of endpoint indicator evaluation. V6: adequacy of follow-up time. V7: loss of follow-up rate <5%. V8: whether sample size was estimated.

### 3.4. Meta-analysis results

#### 3.4.1. Incidence of LS in athletes with LBP.

**Figure 2.**
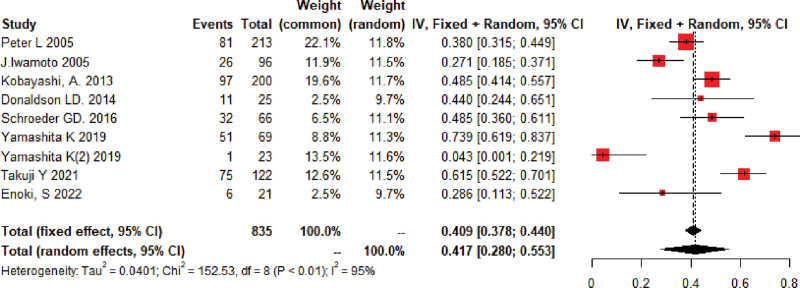
Incidence meta-analysis forest map. Gender 1: Male and female group. Gender 2: Male group.

In the 9 studies^[9,12–20]^ (n = 835) included in this systematic evaluation, there was a large variation in the incidence of LS. The pooled prevalence estimate for the data was 41.7%, [95% CI = (0.28–0.55%)], with significant heterogeneity in the overall results (*I*^2^ = 95%, *P* < .01) (Fig. 2).

**Figure 3.**
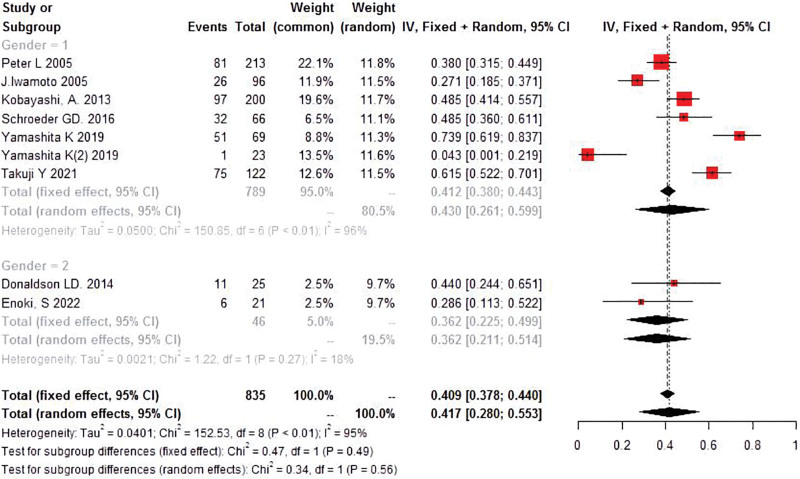
Forest plot of meta-analysis of incidence under different sexes.

**Figure 4.**
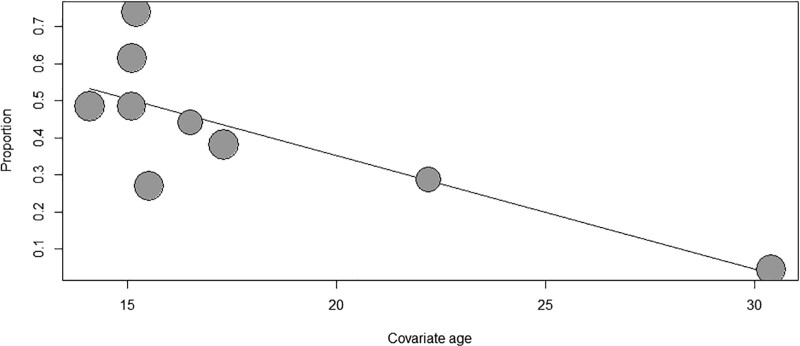
Plot of age-based meta-regression analysis.

Because of the differences in athlete gender between different studies, the gender subgroups discussed the sources of heterogeneity. The results showed that the heterogeneity of male athletes was lower and that of mixed athletes was higher. (Fig. 3). Thus, gender may be a source of heterogeneity. Also, we performed Meta-regression based on the mean age of the patients, and the results showed significant heterogeneity in the incidence of lumbar fissure between ages (PAge = 0.044) (Fig. 4).

**Figure 5.**
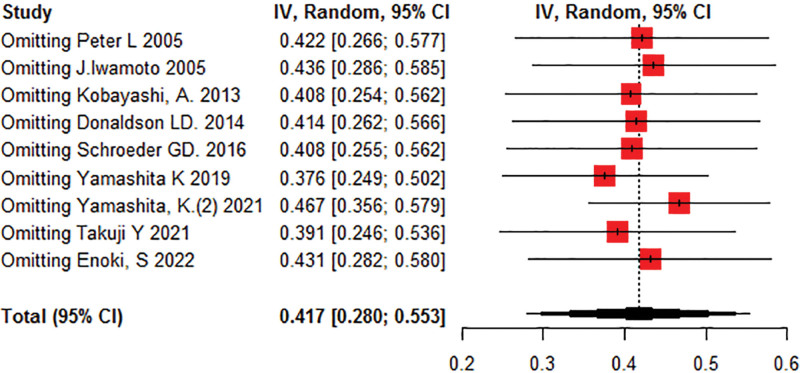
Sensitivity analysis graph for incidence.

Sensitivity analysis showed that the studies included in the analysis were stable, with no significant change in effect size after excluding each study separately (Fig. 5).

#### 3.4.2. Assessment of publication bias.

**Figure 6.**
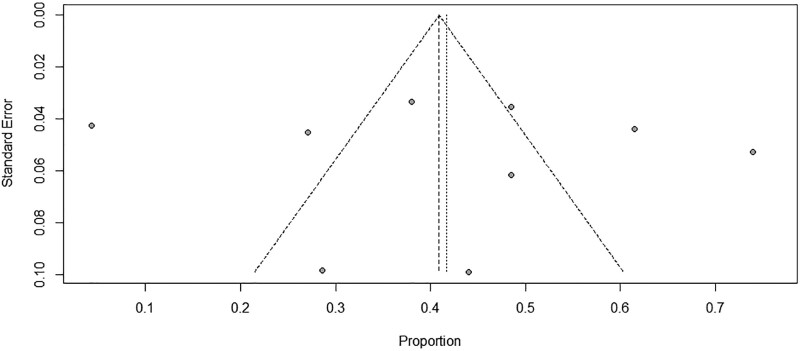
Publication bias plot for incidence.

We used funnel plots to visualize publication bias, and Egger and Begg were used to analyze the funnel plots, and the analysis showed that the corresponding *P* values for the 2 were .85 and .83, respectively. Therefore, it can be concluded that there is no publication bias among studies in terms of pain level relief (Fig. 6).

## 4. Discussion

LBP is a common symptom in athletes, and the incidence of LS is much higher in athletes with LBP than in the general population. Our data summary showed a prevalence estimate of 41.7%, [95% CI = (0.28–0.55)], but this prevalence varied considerably by gender and age of the athletes.

Studies have shown that sports associated with flexion, rotation, and resistance movements of the lumbar spine are more likely to have LS than other sports. Peter L^[12]^ found that the sports with the highest prevalence rates were found in throwing sports (26.67%), artistic gymnastics (16.96%) and rowing (16.88%). J. Iwamoto^[13]^ found that the lumbar spine of rugby players is subjected to significant compressive, shear, and lateral bending loads during play, and theoretically, this dynamic loading pattern places the athletes lumbar motion segments at risk of stressing the intervertebral discs, small joints, and interarticular regions, and this repetitive stress during rugby play may be a factor in the LS and degenerative disc disease (including disc stenosis, spinal instability and small joint disease) the main reason for the high incidence of LBP in athletes. Sugiura S^[21]^ proposed that the mechanism of injury in LS is stress injury caused by repetitive flexion and extension of the spine and trunk rotation. Therefore, this injury is common in sports that require such movements of the spine, including gymnastics, dance, tennis, soccer (side-cutting), weightlifting, and rowing.^[22]^ It’s also been reported in football.^[23]^ In Takuji Y’s study, the enrolled population included soccer, baseball, track and field, and volleyball; however, no significant predictors were found in the type of physical activity.^[18]^ Throwing sports, gymnastics and rowing were described by Soler T^[24]^ as risk factors for LS in elite Spanish athletes, and Sakai T^[25]^ reported the highest incidence of LS in rugby and American soccer players (20.5%). Hangai reported^[26]^ that Japanese collegiate baseball players (59.7%) and swimmers (57.5%) had a significantly higher prevalence of LS than non-16-year-old athletes (31.4%). Therefore, it is conceivable that the pressure on the disc is different and the incidence of LS varies depending on the type of the 18 sport-specific movements, and all orthopedic surgeons should make a comprehensive diagnosis of LS based on the patient’s symptoms, imaging presentation, type of movement, and medical history characteristics.

In the current study, male gender was an independent predictor of positive MRI scan results. In addition, female gender was a negative predictor of vertebral arch cleft in MRI scan results. According to a recent systematic review, male patients were more likely to develop LS than female patients,^[27]^ which is consistent with our results. Some studies report that the prevalence of LS varies by race and gender.^[28]^ Sakai^[29]^ reported a 2:1 male to female ratio of LS patients based on a review of CT scans of 2000 participants aged 20 to 90 years.

Masharawi^[30]^ reported that the wider width of the vertebral interface in girls compared to boys implies better posterior mechanics to resist repetitive sagittal stresses because the area where the spinal load is located is relatively larger. It was also reported^[31]^ that the BMD values of the lumbar spine were significantly higher in girls than in boys, based on the results of a study of 363 healthy children aged 10 to 17 years. These anatomical and biological differences may contribute to the higher incidence of LS in boys than in girls. Stracciolini^[32]^ reported that spondylolisthesis accounts for 50% of spinal injuries in young male athletes, compared to 33.9% of young female athletes with spinal injuries in the United States. Clinicians should consider not only the racial characteristics of LS development in their home country, but also their hospital-specific referral patterns, as these factors may influence the incidence of LS in adolescent athletes.

In our study, there was significant heterogeneity among different age groups in the incidence of LS (PAge = 0.044). The incidence of LS was significantly reduced in the test group with patients aged over 30. Lemoine et al^[33]^ reported that the prevalence of bilateral spondylolysis increased after children have learned to walk. Spondylolysis was observed in 1% of children under the age of 3, in 3.7% of children under age 6, and in 4.7% of children under age 8.

However, Brooks reported that there were 203 positive cases of defects of the lumbar pars interarticularis, with an overall prevalence of 8.0%. Prevalence per decade was fairly evenly distributed and ranged from 7.0% (the age of 30–39 years) to 9.2% (the age of 70 years and above). The prevalence of spondylolysis was 7.9% in patients aged 20 to 49 years, and 8.0% in those aged 50 years and older. Logistic regression showed no significant increase in spondylolysis based on age. Therefore, it is generally recognized that spondylolysis occurs in adolescent under the age of 20 years.^[34]^

Through analysis, we summarized the high risk factors for LS. More frequent physical examination and targeted prophylaxis can be performed for young male athletes who frequently carry out spinal rotation and compression activities. In order to reduce the incidence of LS, it’s advisable to: Add various auxiliary training to reduce the frequency of high-impact spinal activities in addition to necessary training; Avoid stunts and falls on firm ground, and preferably use protective gear during training to protect the spine and avoid stress injuries; Use correct and specific guidelines to perform skills with high technical requirements to avoid unnecessary injuries caused by incorrect actions, inadequate guidance and other factors.^[35,36]^ The first-line treatment for LS is conservative treatment. Adolescent athletes usually respond well to conservative treatment. Symptomatic athletes should avoid hyperextension activities and exercises until the pain disappears, which may take a few days to 6 months.^[37]^ Rehabilitation exercises may include regular hamstring stretches and abdominal core reinforcement exercises in order to improve flexibility.^[38]^

The strength of this study is that it is the first comprehensive meta-analysis of the incidence of LS in athletes with LBP to explore the significance of the incidence of LS in athletes with LBP through an evidence-based approach. However, there are some limitations. First, there are few original studies examining the occurrence of LS in athletes with concomitant LBP; second, the sample sizes of the included studies are generally small; third, the included studies cannot accurately extract the prevalence brought about by a specific sport, etc. The above points cause our study to be somewhat limited, biased, and not very representative. It is hoped that more relevant, high-quality, multicenter, large-sample original studies will be reported in the future, with a refinement of the type of exercise, which I believe will be of great help in exploring the incidence and significance of LS in athletes with LBP.

## 5. Conclusion

The estimated incidence of LS in athletes with LBP was 41.7%, [95% CI = (0.28–0.55)], and future correlations in the incidence of LS in adolescent athletes worldwide need to be evaluated from different perspectives, including biomechanical, hormonal, anatomical, behavioral, and gender differences. Prevention and control interventions should be given promptly and early to athletes in high-risk incidence settings.

## Acknowledgments

I would like to express my heartfelt thanks to Yi Cui for his unconditional trust and support. And I would like to thank all participants in the present study. This study was supported by the Yunnan Orthopedic and Exercise Rehabilitation Clinical Medical Research Center - Bone and soft Tissue Repair and Reconstruction and Spinal Disease Research(202102AA310068); The Science and Technology Plan Project of Yunnan Provincial Department of Science and Technology (202101AY070001-295); Yunnan Orthopedic Trauma Clinical Medical Center (ZX20191001); 920 Hospital Science and Technology Plan Project (2019YGB06).

## Author contributions

**Conceptualization:** Yi Cui.

**Data curation:** Jingyuan Li, Di Du, Fanzhe Feng.

**Formal analysis:** Jingyuan Li, Fanzhe Feng.

**Methodology:** Yongqing Xu, Yi Cui.

**Resources:** Jingyuan Li, Jinlong Liang, Fanzhe Feng.

**Software:** Jinlong Liang, Di Du.

**Supervision:** Yongqing Xu, Di Du.

**Visualization:** Yongqing Xu, Junhong Shen.

**Writing – original draft:** Jingyuan Li, Junhong Shen.

**Writing – review & editing:** Yongqing Xu, Yi Cui.

## Supplementary Material


